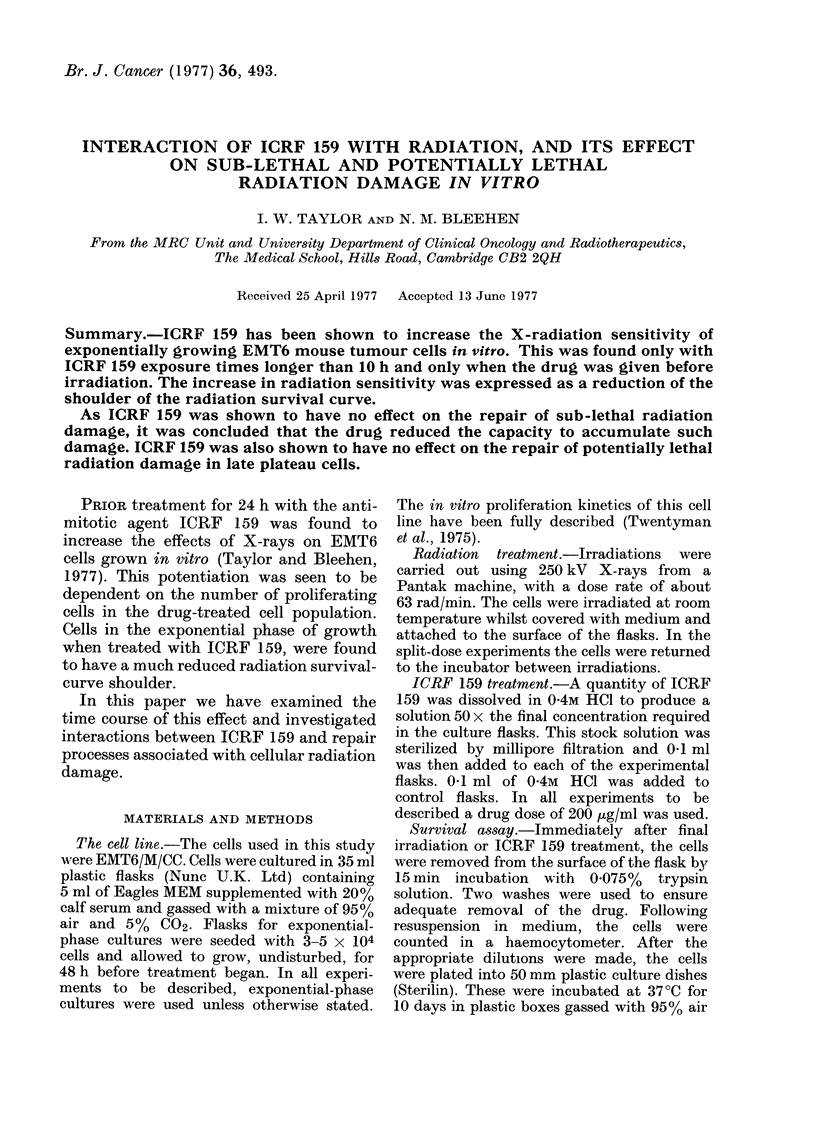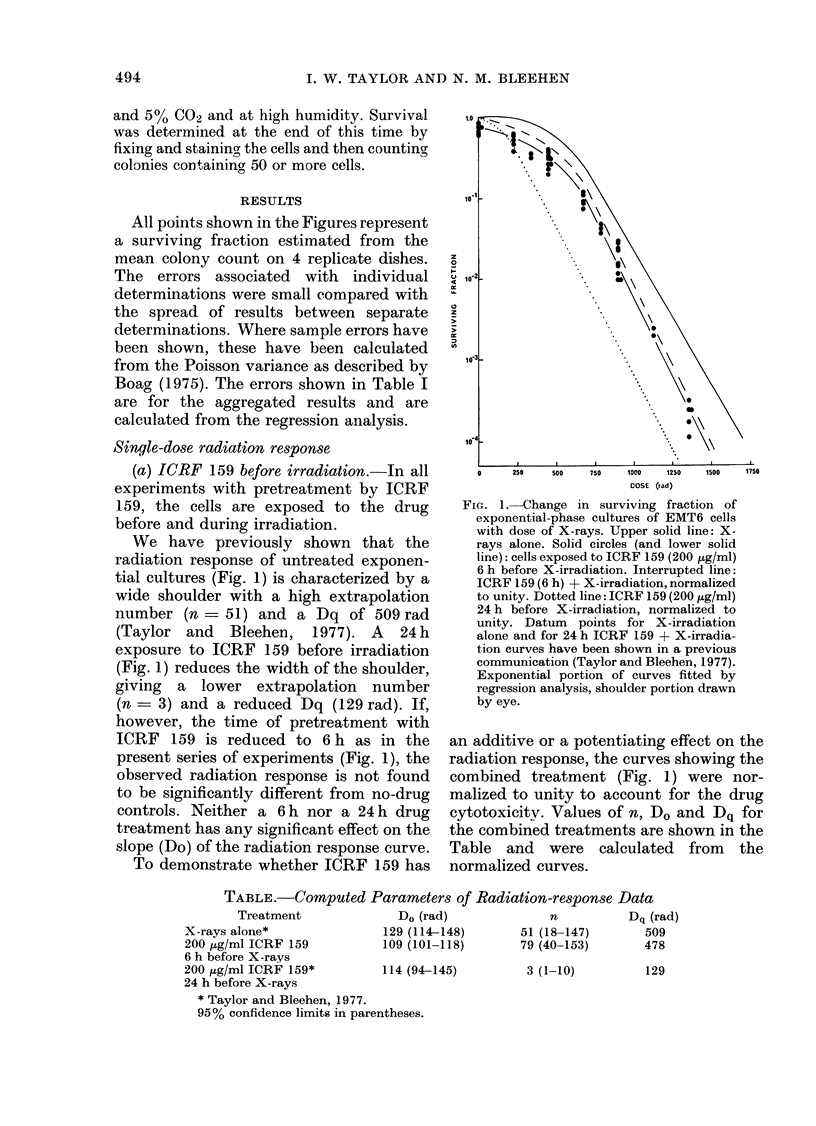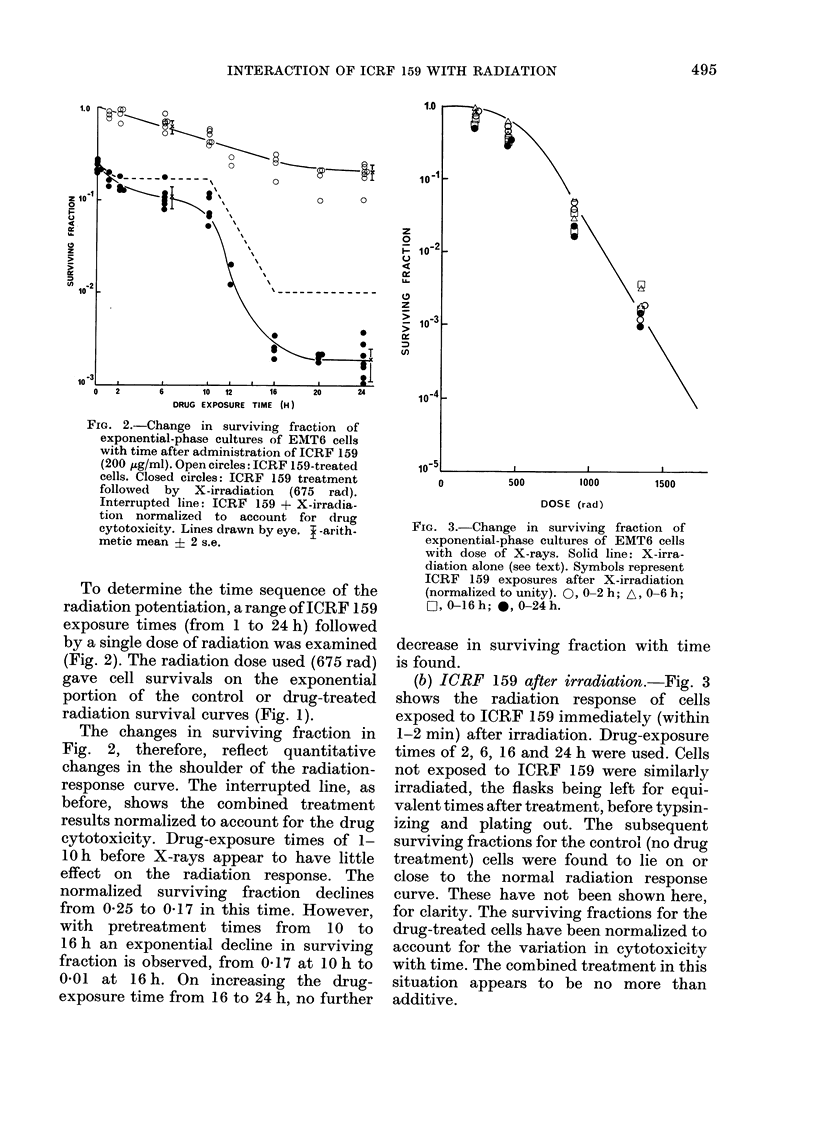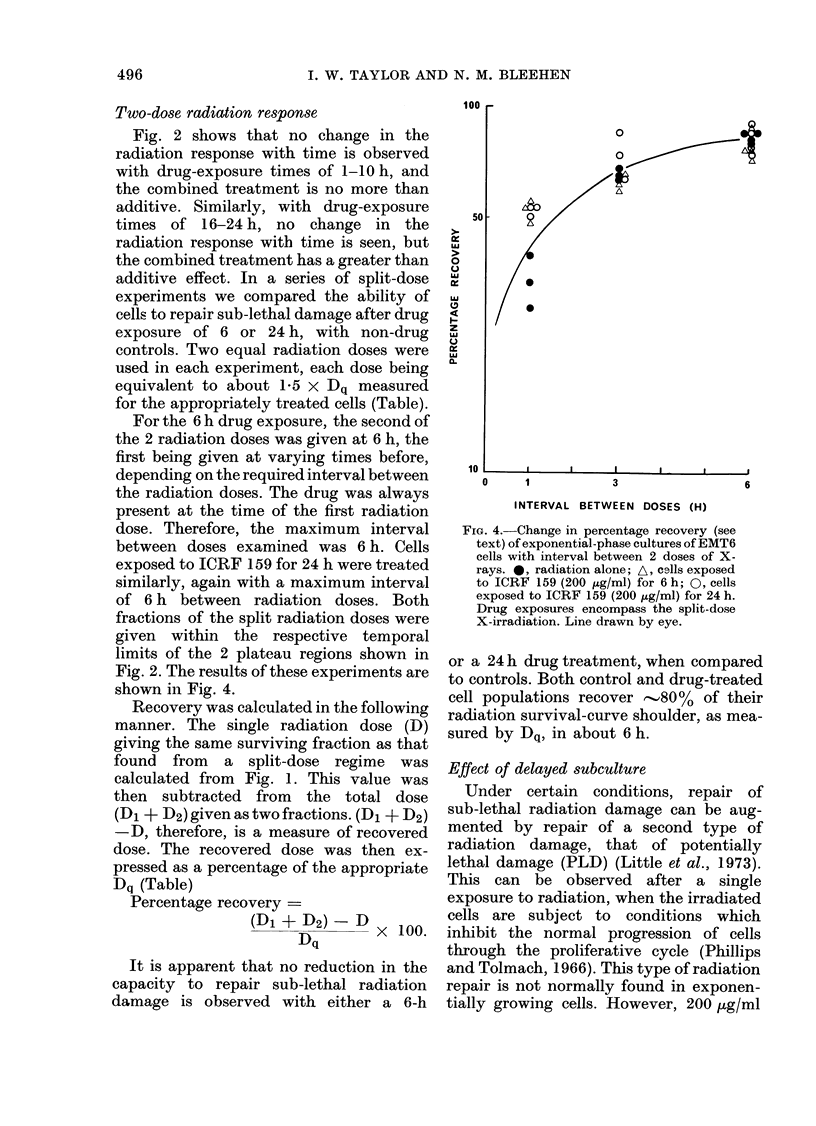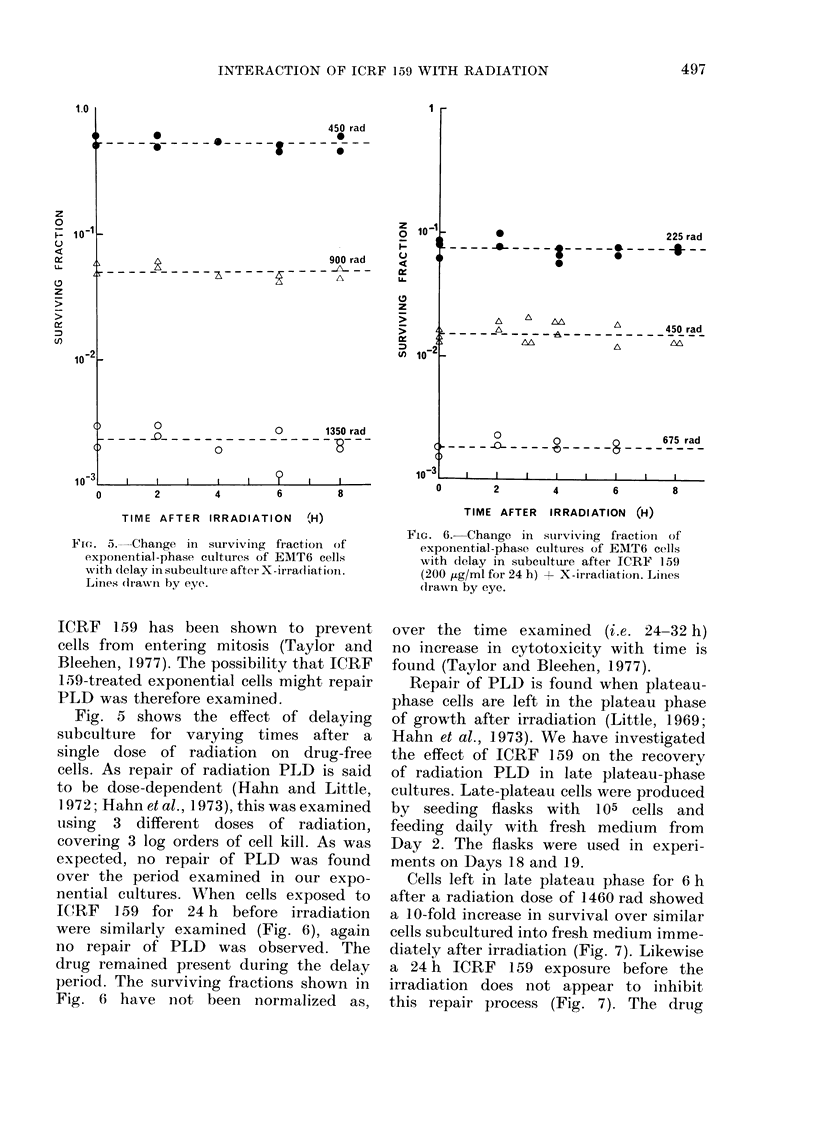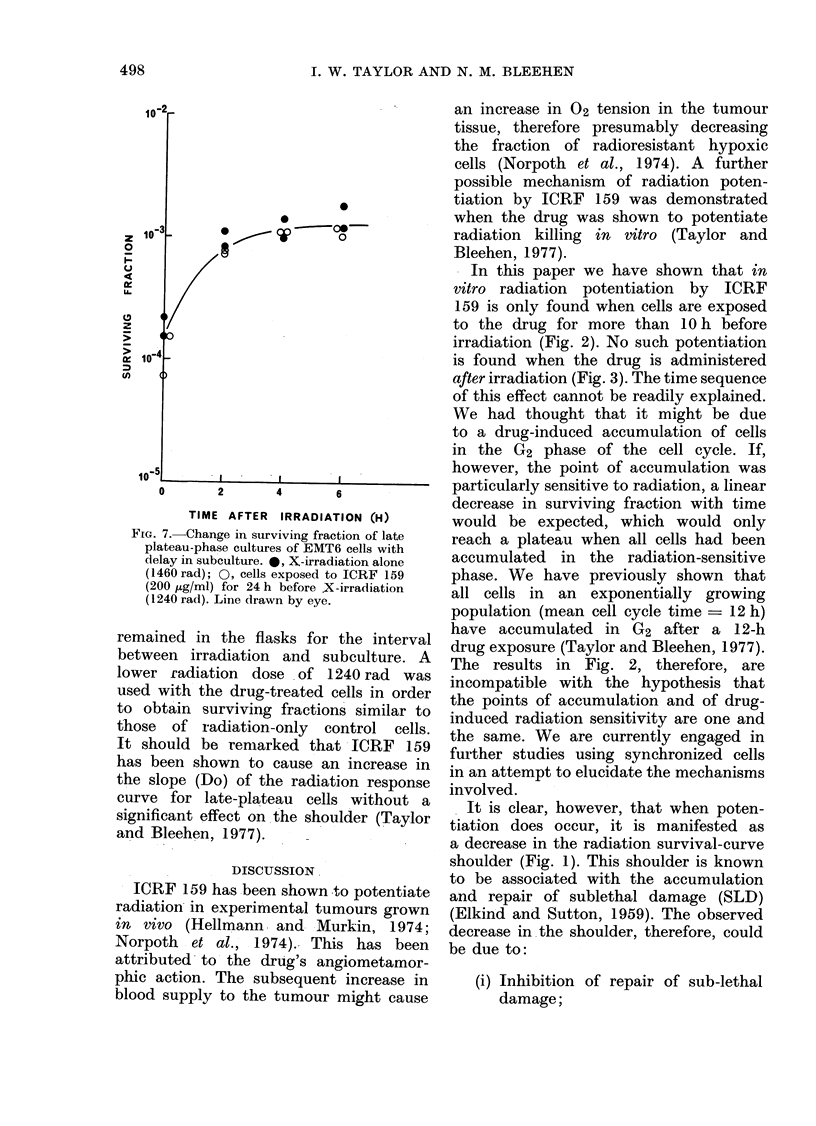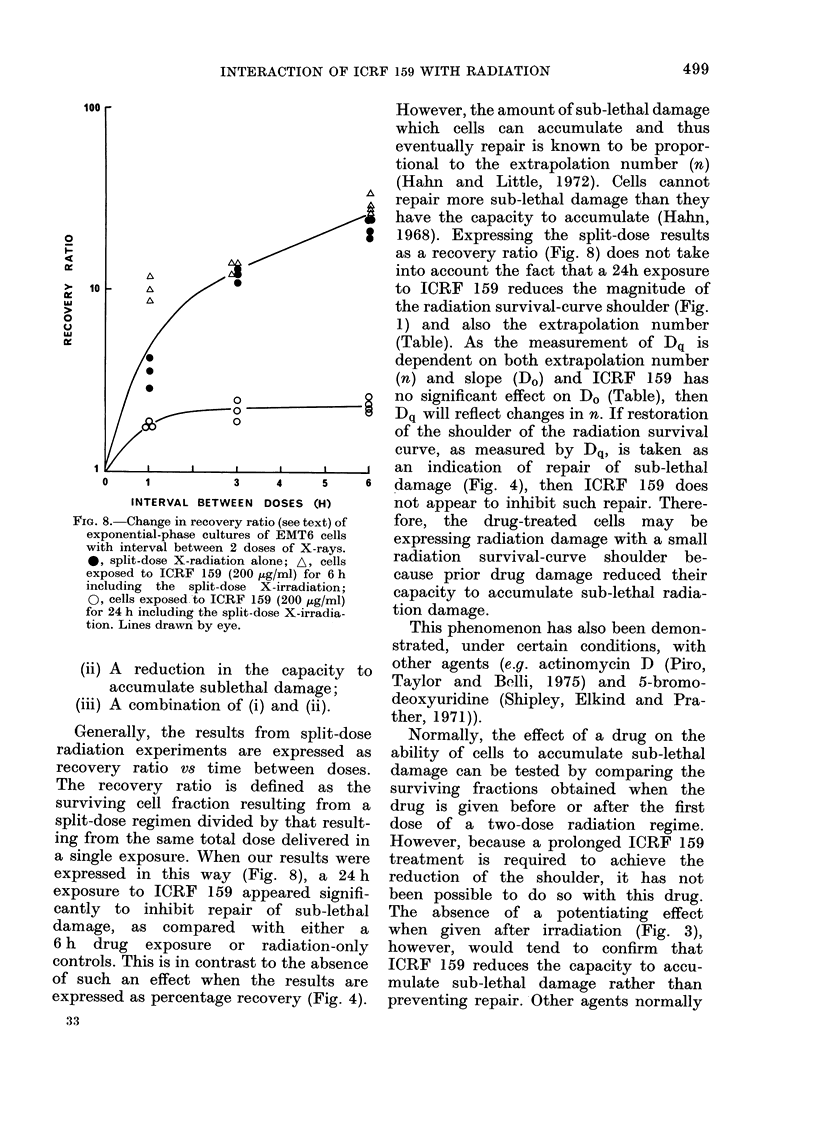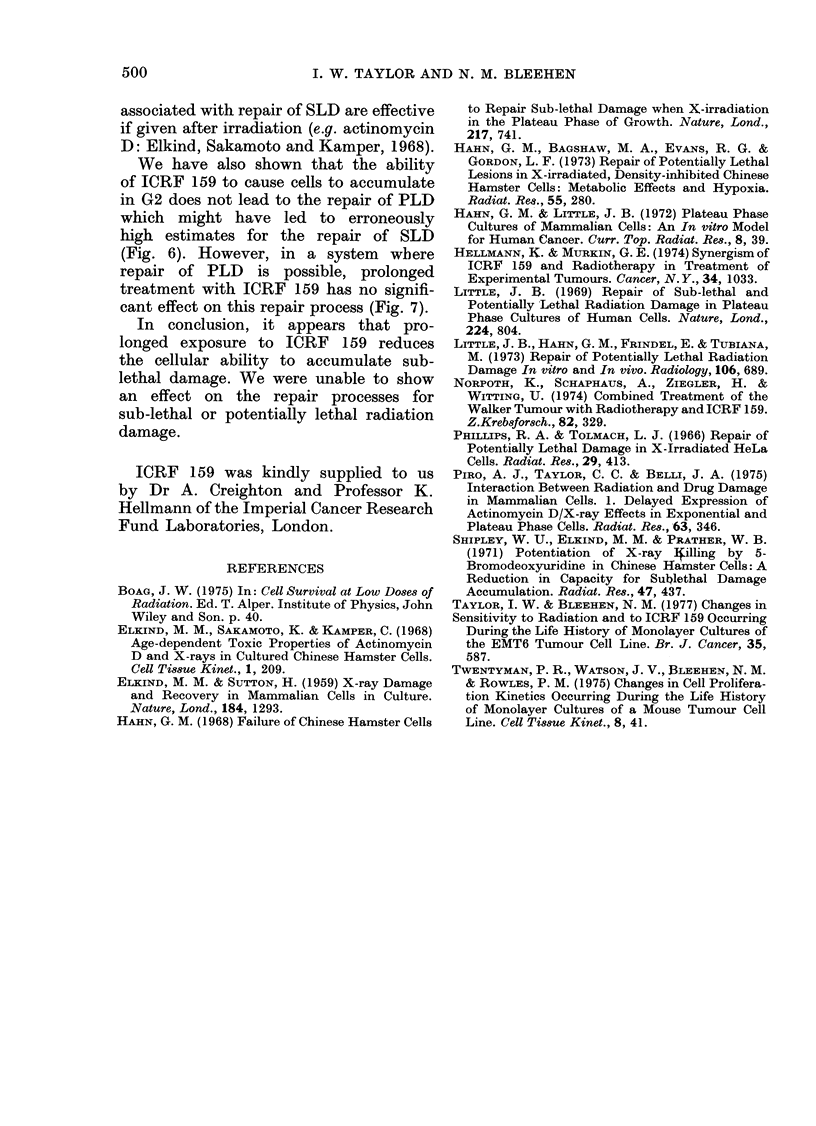# Interaction of ICRF 159 with radiation, and its effect on sub-lethal and potentially lethal radiation damage in vitro.

**DOI:** 10.1038/bjc.1977.219

**Published:** 1977-10

**Authors:** I. W. Taylor, N. M. Bleehen

## Abstract

ICRF 159 has been shown to increase the X-radiation sensitivity of exponentially growing EMT6 mouse tumour cells in vitro. This was found only with ICRF 159 exposure times longer than 10 h and only when the drug was given before irradiation. The increase in radiation sensitivity was expressed as a reduction of the shoulder of the radiation survival curve. As ICRF 159 was shown to have no effect on the repair of sub-lethal radiation damage, it was concluded that the drug reduced the capacity to accumulate such damage. ICRF 159 was also shown to have no effect on the repair of potentially lethal radiation damage in late plateau cells.


*Br. J. Cancer* (1977) **36**, 493.

### INTERACTION OF ICRF 159 WITH RADIATION, AND ITS EFFECT ON SUB-LETHAL AND POTENTIALLY LETHAL RADIATION DAMAGE *IN VITRO*

I. W. TAYLOR AND N. M. BLEEHEN

*From the MRC Unit and University Department of Clinical Oncology and Radiotherapeutics, The Medical School, Hills Road, Cambridge CB2 2QH*

Received 25 April 1977 Accepted 13 June 1977

**Summary.—ICRF 159 has been shown to increase the X-radiation sensitivity of exponentially growing EMT6 mouse tumour cells *in vitro*. This was found only with ICRF 159 exposure times longer than 10 h and only when the drug was given before irradiation. The increase in radiation sensitivity was expressed as a reduction of the shoulder of the radiation survival curve.**

**As ICRF 159 was shown to have no effect on the repair of sub-lethal radiation damage, it was concluded that the drug reduced the capacity to accumulate such damage. ICRF 159 was also shown to have no effect on the repair of potentially lethal radiation damage in late plateau cells.**

PRIOR treatment for 24 h with the anti-mitotic agent ICRF 159 was found to increase the effects of X-rays on EMT6 cells grown *in vitro* (Taylor and Bleehen, 1977). This potentiation was seen to be dependent on the number of proliferating cells in the drug-treated cell population. Cells in the exponential phase of growth when treated with ICRF 159, were found to have a much reduced radiation survival-curve shoulder.

In this paper we have examined the time course of this effect and investigated interactions between ICRF 159 and repair processes associated with cellular radiation damage.

### MATERIALS AND METHODS

*The cell line.*—The cells used in this study were EMT6/M/CC. Cells were cultured in 35 ml plastic flasks (Nunc U.K. Ltd) containing 5 ml of Eagles MEM supplemented with 20% calf serum and gassed with a mixture of 95% air and 5% $CO_2$. Flasks for exponential-phase cultures were seeded with 3–5 $\times 10^4$ cells and allowed to grow, undisturbed, for 48 h before treatment began. In all experiments to be described, exponential-phase cultures were used unless otherwise stated. The *in vitro* proliferation kinetics of this cell line have been fully described (Twentyman *et al.*, 1975).

*Radiation treatment.*—Irradiations were carried out using 250 kV X-rays from a Pantak machine, with a dose rate of about 63 rad/min. The cells were irradiated at room temperature whilst covered with medium and attached to the surface of the flasks. In the split-dose experiments the cells were returned to the incubator between irradiations.

*ICRF* 159 *treatment.*—A quantity of ICRF 159 was dissolved in 0·4M HCl to produce a solution 50 $\times$ the final concentration required in the culture flasks. This stock solution was sterilized by millipore filtration and 0·1 ml was then added to each of the experimental flasks. 0·1 ml of 0·4M HCl was added to control flasks. In all experiments to be described a drug dose of 200 $\mu$g/ml was used.

*Survival assay.*—Immediately after final irradiation or ICRF 159 treatment, the cells were removed from the surface of the flask by 15 min incubation with 0·075% trypsin solution. Two washes were used to ensure adequate removal of the drug. Following resuspension in medium, the cells were counted in a haemocytometer. After the appropriate dilutions were made, the cells were plated into 50 mm plastic culture dishes (Sterilin). These were incubated at 37 °C for 10 days in plastic boxes gassed with 95% air and 5% $CO_2$ and at high humidity. Survival was determined at the end of this time by fixing and staining the cells and then counting colonies containing 50 or more cells.

### RESULTS

All points shown in the Figures represent a surviving fraction estimated from the mean colony count on 4 replicate dishes. The errors associated with individual determinations were small compared with the spread of results between separate determinations. Where sample errors have been shown, these have been calculated from the Poisson variance as described by Boag (1975). The errors shown in Table I are for the aggregated results and are calculated from the regression analysis.

### *Single-dose radiation response*

(*a*) *ICRF* 159 *before irradiation.*—In all experiments with pretreatment by ICRF 159, the cells are exposed to the drug before and during irradiation.

We have previously shown that the radiation response of untreated exponential cultures (Fig. 1) is characterized by a wide shoulder with a high extrapolation number ($n = 51$) and a Dq of 509 rad (Taylor and Bleehen, 1977). A 24 h exposure to ICRF 159 before irradiation (Fig. 1) reduces the width of the shoulder, giving a lower extrapolation number ($n = 3$) and a reduced Dq (129 rad). If, however, the time of pretreatment with ICRF 159 is reduced to 6 h as in the present series of experiments (Fig. 1), the observed radiation response is not found to be significantly different from no-drug controls. Neither a 6 h nor a 24 h drug treatment has any significant effect on the slope (Do) of the radiation response curve.

To demonstrate whether ICRF 159 has

FIG. 1.—Change in surviving fraction of exponential-phase cultures of EMT6 cells with dose of X-rays. Upper solid line: X-rays alone. Solid circles (and lower solid line): cells exposed to ICRF 159 (200 μg/ml) 6 h before X-irradiation. Interrupted line: ICRF 159 (6 h) + X-irradiation, normalized to unity. Dotted line: ICRF 159 (200 μg/ml) 24 h before X-irradiation, normalized to unity. Datum points for X-irradiation alone and for 24 h ICRF 159 + X-irradiation curves have been shown in a previous communication (Taylor and Bleehen, 1977). Exponential portion of curves fitted by regression analysis, shoulder portion drawn by eye.

an additive or a potentiating effect on the radiation response, the curves showing the combined treatment (Fig. 1) were normalized to unity to account for the drug cytotoxicity. Values of $n$, $D_0$ and $D_q$ for the combined treatments are shown in the Table and were calculated from the normalized curves.

TABLE.—*Computed Parameters of Radiation-response Data*

| Treatment | $D_0$ (rad) | $n$ | $D_q$ (rad) |
|---|---|---|---|
| X-rays alone* | 129 (114–148) | 51 (18–147) | 509 |
| 200 μg/ml ICRF 159 6 h before X-rays | 109 (101–118) | 79 (40–153) | 478 |
| 200 μg/ml ICRF 159* 24 h before X-rays | 114 (94–145) | 3 (1–10) | 129 |

* Taylor and Bleehen, 1977.
95% confidence limits in parentheses.

FIG. 2.—Change in surviving fraction of exponential-phase cultures of EMT6 cells with time after administration of ICRF 159 (200 μg/ml). Open circles: ICRF 159-treated cells. Closed circles: ICRF 159 treatment followed by X-irradiation (675 rad). Interrupted line: ICRF 159 + X-irradiation normalized to account for drug cytotoxicity. Lines drawn by eye. Ī-arithmetic mean ± 2 s.e.

FIG. 3.—Change in surviving fraction of exponential-phase cultures of EMT6 cells with dose of X-rays. Solid line: X-irradiation alone (see text). Symbols represent ICRF 159 exposures after X-irradiation (normalized to unity). ○, 0–2 h; △, 0–6 h; □, 0–16 h; ●, 0–24 h.

To determine the time sequence of the radiation potentiation, a range of ICRF 159 exposure times (from 1 to 24 h) followed by a single dose of radiation was examined (Fig. 2). The radiation dose used (675 rad) gave cell survivals on the exponential portion of the control or drug-treated radiation survival curves (Fig. 1).

The changes in surviving fraction in Fig. 2, therefore, reflect quantitative changes in the shoulder of the radiation-response curve. The interrupted line, as before, shows the combined treatment results normalized to account for the drug cytotoxicity. Drug-exposure times of 1–10 h before X-rays appear to have little effect on the radiation response. The normalized surviving fraction declines from 0·25 to 0·17 in this time. However, with pretreatment times from 10 to 16 h an exponential decline in surviving fraction is observed, from 0·17 at 10 h to 0·01 at 16 h. On increasing the drug-exposure time from 16 to 24 h, no further decrease in surviving fraction with time is found.

(*b*) *ICRF* 159 *after irradiation.*—Fig. 3 shows the radiation response of cells exposed to ICRF 159 immediately (within 1–2 min) after irradiation. Drug-exposure times of 2, 6, 16 and 24 h were used. Cells not exposed to ICRF 159 were similarly irradiated, the flasks being left for equivalent times after treatment, before typsinizing and plating out. The subsequent surviving fractions for the control (no drug treatment) cells were found to lie on or close to the normal radiation response curve. These have not been shown here, for clarity. The surviving fractions for the drug-treated cells have been normalized to account for the variation in cytotoxicity with time. The combined treatment in this situation appears to be no more than additive.

*Two-dose radiation response*

Fig. 2 shows that no change in the radiation response with time is observed with drug-exposure times of 1–10 h, and the combined treatment is no more than additive. Similarly, with drug-exposure times of 16–24 h, no change in the radiation response with time is seen, but the combined treatment has a greater than additive effect. In a series of split-dose experiments we compared the ability of cells to repair sub-lethal damage after drug exposure of 6 or 24 h, with non-drug controls. Two equal radiation doses were used in each experiment, each dose being equivalent to about 1·5 × $D_q$ measured for the appropriately treated cells (Table).

For the 6 h drug exposure, the second of the 2 radiation doses was given at 6 h, the first being given at varying times before, depending on the required interval between the radiation doses. The drug was always present at the time of the first radiation dose. Therefore, the maximum interval between doses examined was 6 h. Cells exposed to ICRF 159 for 24 h were treated similarly, again with a maximum interval of 6 h between radiation doses. Both fractions of the split radiation doses were given within the respective temporal limits of the 2 plateau regions shown in Fig. 2. The results of these experiments are shown in Fig. 4.

Recovery was calculated in the following manner. The single radiation dose (D) giving the same surviving fraction as that found from a split-dose regime was calculated from Fig. 1. This value was then subtracted from the total dose ($D_1 + D_2$) given as two fractions. ($D_1 + D_2$) —D, therefore, is a measure of recovered dose. The recovered dose was then expressed as a percentage of the appropriate $D_q$ (Table)

$$\text{Percentage recovery} = \frac{(D_1 + D_2) - D}{D_q} \times 100.$$

It is apparent that no reduction in the capacity to repair sub-lethal radiation damage is observed with either a 6-h

Fig. 4.—Change in percentage recovery (see text) of exponential-phase cultures of EMT6 cells with interval between 2 doses of X-rays. ●, radiation alone; △, cells exposed to ICRF 159 (200 μg/ml) for 6 h; ○, cells exposed to ICRF 159 (200 μg/ml) for 24 h. Drug exposures encompass the split-dose X-irradiation. Line drawn by eye.

or a 24 h drug treatment, when compared to controls. Both control and drug-treated cell populations recover ~80% of their radiation survival-curve shoulder, as measured by $D_q$, in about 6 h.

*Effect of delayed subculture*

Under certain conditions, repair of sub-lethal radiation damage can be augmented by repair of a second type of radiation damage, that of potentially lethal damage (PLD) (Little *et al.*, 1973). This can be observed after a single exposure to radiation, when the irradiated cells are subject to conditions which inhibit the normal progression of cells through the proliferative cycle (Phillips and Tolmach, 1966). This type of radiation repair is not normally found in exponentially growing cells. However, 200 μg/ml

FIG. 5.—Change in surviving fraction of exponential-phase cultures of EMT6 cells with delay in subculture after X-irradiation. Lines drawn by eye.

FIG. 6.—Change in surviving fraction of exponential-phase cultures of EMT6 cells with delay in subculture after ICRF 159 (200 $\mu$g/ml for 24 h) + X-irradiation. Lines drawn by eye.

ICRF 159 has been shown to prevent cells from entering mitosis (Taylor and Bleehen, 1977). The possibility that ICRF 159-treated exponential cells might repair PLD was therefore examined.

Fig. 5 shows the effect of delaying subculture for varying times after a single dose of radiation on drug-free cells. As repair of radiation PLD is said to be dose-dependent (Hahn and Little, 1972; Hahn *et al.*, 1973), this was examined using 3 different doses of radiation, covering 3 log orders of cell kill. As was expected, no repair of PLD was found over the period examined in our exponential cultures. When cells exposed to ICRF 159 for 24 h before irradiation were similarly examined (Fig. 6), again no repair of PLD was observed. The drug remained present during the delay period. The surviving fractions shown in Fig. 6 have not been normalized as, over the time examined (*i.e.* 24–32 h) no increase in cytotoxicity with time is found (Taylor and Bleehen, 1977).

Repair of PLD is found when plateau-phase cells are left in the plateau phase of growth after irradiation (Little, 1969; Hahn *et al.*, 1973). We have investigated the effect of ICRF 159 on the recovery of radiation PLD in late plateau-phase cultures. Late-plateau cells were produced by seeding flasks with $10^5$ cells and feeding daily with fresh medium from Day 2. The flasks were used in experiments on Days 18 and 19.

Cells left in late plateau phase for 6 h after a radiation dose of 1460 rad showed a 10-fold increase in survival over similar cells subcultured into fresh medium immediately after irradiation (Fig. 7). Likewise a 24 h ICRF 159 exposure before the irradiation does not appear to inhibit this repair process (Fig. 7). The drug

FIG. 7.—Change in surviving fraction of late plateau-phase cultures of EMT6 cells with delay in subculture. ●, X-irradiation alone (1460 rad); ○, cells exposed to ICRF 159 (200 μg/ml) for 24 h before X-irradiation (1240 rad). Line drawn by eye.

remained in the flasks for the interval between irradiation and subculture. A lower radiation dose of 1240 rad was used with the drug-treated cells in order to obtain surviving fractions similar to those of radiation-only control cells. It should be remarked that ICRF 159 has been shown to cause an increase in the slope (Do) of the radiation response curve for late-plateau cells without a significant effect on the shoulder (Taylor and Bleehen, 1977).

### DISCUSSION

ICRF 159 has been shown to potentiate radiation in experimental tumours grown *in vivo* (Hellmann and Murkin, 1974; Norpoth *et al.*, 1974). This has been attributed to the drug's angiometamorphic action. The subsequent increase in blood supply to the tumour might cause an increase in $O_2$ tension in the tumour tissue, therefore presumably decreasing the fraction of radioresistant hypoxic cells (Norpoth *et al.*, 1974). A further possible mechanism of radiation potentiation by ICRF 159 was demonstrated when the drug was shown to potentiate radiation killing *in vitro* (Taylor and Bleehen, 1977).

In this paper we have shown that *in vitro* radiation potentiation by ICRF 159 is only found when cells are exposed to the drug for more than 10 h before irradiation (Fig. 2). No such potentiation is found when the drug is administered *after* irradiation (Fig. 3). The time sequence of this effect cannot be readily explained. We had thought that it might be due to a drug-induced accumulation of cells in the $G_2$ phase of the cell cycle. If, however, the point of accumulation was particularly sensitive to radiation, a linear decrease in surviving fraction with time would be expected, which would only reach a plateau when all cells had been accumulated in the radiation-sensitive phase. We have previously shown that all cells in an exponentially growing population (mean cell cycle time = 12 h) have accumulated in $G_2$ after a 12-h drug exposure (Taylor and Bleehen, 1977). The results in Fig. 2, therefore, are incompatible with the hypothesis that the points of accumulation and of drug-induced radiation sensitivity are one and the same. We are currently engaged in further studies using synchronized cells in an attempt to elucidate the mechanisms involved.

It is clear, however, that when potentiation does occur, it is manifested as a decrease in the radiation survival-curve shoulder (Fig. 1). This shoulder is known to be associated with the accumulation and repair of sublethal damage (SLD) (Elkind and Sutton, 1959). The observed decrease in the shoulder, therefore, could be due to:

(i) Inhibition of repair of sub-lethal damage;

FIG. 8.—Change in recovery ratio (see text) of exponential-phase cultures of EMT6 cells with interval between 2 doses of X-rays. ●, split-dose X-radiation alone; △, cells exposed to ICRF 159 (200 μg/ml) for 6 h including the split-dose X-irradiation; ○, cells exposed to ICRF 159 (200 μg/ml) for 24 h including the split-dose X-irradiation. Lines drawn by eye.

(ii) A reduction in the capacity to accumulate sublethal damage;
(iii) A combination of (i) and (ii).

Generally, the results from split-dose radiation experiments are expressed as recovery ratio *vs* time between doses. The recovery ratio is defined as the surviving cell fraction resulting from a split-dose regimen divided by that resulting from the same total dose delivered in a single exposure. When our results were expressed in this way (Fig. 8), a 24 h exposure to ICRF 159 appeared significantly to inhibit repair of sub-lethal damage, as compared with either a 6 h drug exposure or radiation-only controls. This is in contrast to the absence of such an effect when the results are expressed as percentage recovery (Fig. 4). However, the amount of sub-lethal damage which cells can accumulate and thus eventually repair is known to be proportional to the extrapolation number (*n*) (Hahn and Little, 1972). Cells cannot repair more sub-lethal damage than they have the capacity to accumulate (Hahn, 1968). Expressing the split-dose results as a recovery ratio (Fig. 8) does not take into account the fact that a 24h exposure to ICRF 159 reduces the magnitude of the radiation survival-curve shoulder (Fig. 1) and also the extrapolation number (Table). As the measurement of $D_q$ is dependent on both extrapolation number (*n*) and slope ($D_0$) and ICRF 159 has no significant effect on $D_0$ (Table), then $D_q$ will reflect changes in *n*. If restoration of the shoulder of the radiation survival curve, as measured by $D_q$, is taken as an indication of repair of sub-lethal damage (Fig. 4), then ICRF 159 does not appear to inhibit such repair. Therefore, the drug-treated cells may be expressing radiation damage with a small radiation survival-curve shoulder because prior drug damage reduced their capacity to accumulate sub-lethal radiation damage.

This phenomenon has also been demonstrated, under certain conditions, with other agents (*e.g.* actinomycin D (Piro, Taylor and Belli, 1975) and 5-bromodeoxyuridine (Shipley, Elkind and Prather, 1971)).

Normally, the effect of a drug on the ability of cells to accumulate sub-lethal damage can be tested by comparing the surviving fractions obtained when the drug is given before or after the first dose of a two-dose radiation regime. However, because a prolonged ICRF 159 treatment is required to achieve the reduction of the shoulder, it has not been possible to do so with this drug. The absence of a potentiating effect when given after irradiation (Fig. 3), however, would tend to confirm that ICRF 159 reduces the capacity to accumulate sub-lethal damage rather than preventing repair. Other agents normally associated with repair of SLD are effective if given after irradiation (*e.g.* actinomycin D: Elkind, Sakamoto and Kamper, 1968).

We have also shown that the ability of ICRF 159 to cause cells to accumulate in G2 does not lead to the repair of PLD which might have led to erroneously high estimates for the repair of SLD (Fig. 6). However, in a system where repair of PLD is possible, prolonged treatment with ICRF 159 has no significant effect on this repair process (Fig. 7).

In conclusion, it appears that prolonged exposure to ICRF 159 reduces the cellular ability to accumulate sub-lethal damage. We were unable to show an effect on the repair processes for sub-lethal or potentially lethal radiation damage.

ICRF 159 was kindly supplied to us by Dr A. Creighton and Professor K. Hellmann of the Imperial Cancer Research Fund Laboratories, London.

### REFERENCES

BOAG, J. W. (1975) In: *Cell Survival at Low Doses of Radiation*. Ed. T. Alper. Institute of Physics, John Wiley and Son. p. 40.

ELKIND, M. M., SAKAMOTO, K. & KAMPER, C. (1968) Age-dependent Toxic Properties of Actinomycin D and X-rays in Cultured Chinese Hamster Cells. *Cell Tissue Kinet.*, **1**, 209.

ELKIND, M. M. & SUTTON, H. (1959) X-ray Damage and Recovery in Mammalian Cells in Culture. *Nature, Lond.*, **184**, 1293.

HAHN, G. M. (1968) Failure of Chinese Hamster Cells to Repair Sub-lethal Damage when X-irradiation in the Plateau Phase of Growth. *Nature, Lond.*, **217**, 741.

HAHN, G. M., BAGSHAW, M. A., EVANS, R. G. & GORDON, L. F. (1973) Repair of Potentially Lethal Lesions in X-irradiated, Density-inhibited Chinese Hamster Cells: Metabolic Effects and Hypoxia. *Radiat. Res.*, **55**, 280.

HAHN, G. M. & LITTLE, J. B. (1972) Plateau Phase Cultures of Mammalian Cells: An *In vitro* Model for Human Cancer. *Curr. Top. Radiat. Res.*, **8**, 39.

HELLMANN, K. & MURKIN, G. E. (1974) Synergism of ICRF 159 and Radiotherapy in Treatment of Experimental Tumours. *Cancer, N.Y.*, **34**, 1033.

LITTLE, J. B. (1969) Repair of Sub-lethal and Potentially Lethal Radiation Damage in Plateau Phase Cultures of Human Cells. *Nature, Lond.*, **224**, 804.

LITTLE, J. B., HAHN, G. M., FRINDEL, E. & TUBIANA, M. (1973) Repair of Potentially Lethal Radiation Damage *In vitro* and *In vivo*. *Radiology*, **106**, 689.

NORPOTH, K., SCHAPHAUS, A., ZIEGLER, H. & WITTING, U. (1974) Combined Treatment of the Walker Tumour with Radiotherapy and ICRF 159. *Z.Krebsforsch.*, **82**, 329.

PHILLIPS, R. A. & TOLMACH, L. J. (1966) Repair of Potentially Lethal Damage in X-Irradiated HeLa Cells. *Radiat. Res.*, **29**, 413.

PIRO, A. J., TAYLOR, C. C. & BELLI, J. A. (1975) Interaction Between Radiation and Drug Damage in Mammalian Cells. 1. Delayed Expression of Actinomycin D/X-ray Effects in Exponential and Plateau Phase Cells. *Radiat. Res.*, **63**, 346.

SHIPLEY, W. U., ELKIND, M. M. & PRATHER, W. B. (1971) Potentiation of X-ray Killing by 5-Bromodeoxyuridine in Chinese Hamster Cells: A Reduction in Capacity for Sublethal Damage Accumulation. *Radiat. Res.*, **47**, 437.

TAYLOR, I. W. & BLEEHEN, N. M. (1977) Changes in Sensitivity to Radiation and to ICRF 159 Occurring During the Life History of Monolayer Cultures of the EMT6 Tumour Cell Line. *Br. J. Cancer*, **35**, 587.

TWENTYMAN, P. R., WATSON, J. V., BLEEHEN, N. M. & ROWLES, P. M. (1975) Changes in Cell Proliferation Kinetics Occurring During the Life History of Monolayer Cultures of a Mouse Tumour Cell Line. *Cell Tissue Kinet.*, **8**, 41.